# Experience with burdens of diabetes device use that affect uptake and optimal use in people with type 1 diabetes

**DOI:** 10.1530/EC-23-0193

**Published:** 2023-09-08

**Authors:** Molly L Tanenbaum, Persis V Commissariat

**Affiliations:** 1 Division of Endocrinology, Gerontology, and Metabolism, Department of Medicine, Stanford University School of Medicine, Stanford, California, USA; 2 Section on Clinical, Behavioral, and Outcomes Research, Joslin Diabetes Center, Boston, Massachusetts, USA

**Keywords:** insulin pumps, continuous glucose monitors, CGM, automated insulin delivery, diabetes distress, patient perspective, acceptance, burden

## Abstract

Diabetes technology continues to advance, with more individuals with type 1 diabetes (T1D) adopting insulin pumps, continuous glucose monitoring (CGM), and automated insulin delivery (AID) systems that integrate real-time glucose data with an algorithm to assist with insulin dosing decisions. These technologies are linked with benefits to glycemic outcomes (e.g. increased time in target range), diabetes management behaviors, and quality of life. However, current devices and systems are not without barriers and hassles for the user. The intent of this review is to describe the personal challenges and reactions that users experience when interacting with current diabetes technologies, which can affect their acceptance and motivation to engage with their devices. This review will discuss user experiences and strategies to address three main areas: (i) the emotional burden of utilizing a wearable device; (ii) the perceived and experienced negative social consequences of device use; and (iii) the practical challenges of wearing devices.

## Introduction

Diabetes devices are rapidly advancing in their features and functionalities, offering a range of benefits for individuals with type 1 diabetes who opt to use this technology. Current diabetes technology includes insulin pumps; continuous glucose monitoring devices (CGM); and automated insulin delivery systems (AID) that combine the pump and CGM functions through an algorithm that can assist with regulating glucose levels. In the past 6 years, five distinct AID systems received FDA approval and are available to the public, demonstrating significant benefits to time-in-range and other glycemic outcomes (1, 3, 4, 5, 6, 8; https://diatribe.org/omnipod-5-approved-fda). This year, an additional do-it-yourself AID system was also cleared for use in the US for the first time (https://diatribe.org/tidepool-loop-cleared-fda). Additionally, two new CGM models were released in the US (https://www.jdrf.org/blog/2022/12/08/dexcom-g7-continuous-glucose-monitor-cleared-fda/; https://www.jdrf.org/blog/2022/05/31/fda-approved-abbott-freestyle-libre-3-continuous-glucose-monitor-ages-4/). Large US, European, and international registry studies have found that compared to non-users of CGM, CGM users had fewer severe hypoglycemic events, fewer episodes of diabetic ketoacidosis (DKA), and better overall diabetes management (as assessed through HbA1c) (8, 9, 10, 11). These advanced technologies have also shown benefits in improving diabetes distress and quality of life (12, 13, 14, 15), and the benefits to the user will likely continue to grow and evolve as technology advances. However, many will continue to experience known barriers and hassles associated with device use (16, 17, 18), as well as potential new challenges to using new device features and functions. It is important to understand the scope of challenges that users perceive and experience when using devices, as the everyday burdens of device use, and the emotional experience of those burdens, will likely drive decisions to start diabetes devices (*uptake*); to use devices over time (*sustained use*); and to use diabetes devices most effectively (*optimal use*). Users will engage in a personal cost–benefit analysis of whether devices are worthwhile to use prior to uptake and throughout use, whether actively or subconsciously, as each experience with technology contributes to their overall impression of their devices.

In this review, we will examine current literature on patient-reported, everyday challenges to diabetes device uptake and sustained/optimal use and discuss potential approaches to address these burdens. We have organized challenges into three overarching areas: (i) the emotional burden of utilizing a wearable device; (ii) the perceived and experienced negative social consequences of device use; and (iii) the practical challenges of wearing devices. Regardless of the type of challenge that occurs, users will inevitably experience a personal reaction that contributes to their thought process around using devices. Thus, this review emphasizes the potential feelings or emotional reactions that users may experience in response to each barrier they face.

## Methods

We conducted a narrative review of the literature on barriers, burdens, and challenges to the uptake and use of diabetes technologies. We also searched the literature on processes to increase technology uptake, sustained use, and optimal use. Given the rapidly changing landscape of diabetes technologies, this review emphasizes research published within the last 5 years.

## Emotional burdens

Diabetes technologies all require attention, effort, and adaptation to unplanned management needs to ensure that devices and systems are working optimally. The stress of managing devices can lead to a significant emotional burden in people with T1D. For some, negative emotional responses to device use can be specific to device features and functionalities (e.g. excessive alerts). Others may perceive devices as part of the greater burden of living with diabetes, affecting their well-being. Emotional challenges likely have a cyclical nature with device uptake and use, as negative emotions can affect motivation and engagement, which in turn can affect the efficacy of the devices and which may cause more emotional burden in needing to troubleshoot issues.

Day-to-day challenges of diabetes technologies can elicit negative emotional responses. All diabetes technologies require user interaction with the system, from placing the site on the body to planned management behaviors to troubleshooting unplanned issues with devices. The need for constant interaction with the system can be emotionally taxing. Users have described devices as a physical reminder of having diabetes (19, 20) and their upkeep disruptive to daily activities at times (21). Continuous glucose monitoring and AID systems also make glucose data easily accessible to the user. This can be a source of emotional burden, as large amounts of data can lead to feeling overwhelmed by ‘data overload’ (22). Users and caregivers report challenges with navigating data in real-time and/or retrospectively to identify patterns and make dosing adjustments (19, 23, 24). Multiple studies have noted responding to alerts and alarms as a significant disruption and source of frustration for users (20, 21, 25, 26, 27). While alerts offer a safety net for users by warning them of out-of-range glucose levels or device issues (e.g. signal loss and site failure), users have suggested that alarms can evoke strong negative emotional responses in people (e.g. anger, frustration, and panic) (25) or feelings of failure in not achieving target glucose levels (23). They can also contribute to sleep disruptions (15). Notably, unexpected alerts can be particularly frustrating because they require users to engage in ‘extra,’ unplanned technology management (28). Alerts that feel disruptive or excessive can lead to ‘alarm fatigue,’ in which people become desensitized and respond less to alert sounds and may discontinue use (29).

Body image concerns may further impede the use of devices that must be worn on the body (30, 31). A large national survey of 1503 adults with T1D conducted through the Type 1 Diabetes Exchange (T1DX) clinic registry found that 26% of adults reported they did not ‘like how diabetes devices look on my body’ (16). Another study of adults using CGM identified body image concerns around device use because people felt self-conscious about a device on the body and viewed it as a physical announcement of their diabetes (19).

Importantly, perceptions play an important role in the emotional challenges of using devices. People may be more likely to start and sustain the use of diabetes devices if they have realistic expectations (32), have trust in the accuracy and reliability of devices (33), and have a perception of the benefits of their devices for their own lives (34). Perceptions of usefulness and ease of use have been highlighted as key indicators of device uptake and sustained use (35). Diabetes management is already demanding; it is possible that when challenges outweigh the benefits of technology, users experience greater emotional distress around diabetes care and may discontinue use. Much research suggests that devices are associated with reduced distress in people with diabetes (36, 37, 38), while others suggest devices may contribute to the overall burden of diabetes (39). Studies of adults in the T1DX Registry as well as youth and adults in Germany and the Netherlands have identified the presence of elevated distress regardless of if/what technology is used (40, 41, 42); however, those who report more barriers to technology use have also been found to endorse greater distress (16). Age and life stage can also play a role in the acceptance and use of diabetes devices. Older adults may experience different priorities, benefits, and barriers when engaging with diabetes devices (43) compared to youth, young adults, or parents of children with T1D.

The relationship between diabetes devices and distress is likely influenced by person-specific factors, such as expectations of devices, perceptions of technology, and (good and bad) experiences with devices. For example, financial burden is a significant concern for people with diabetes as it affects access to supplies (44, 45, 46), and people who experience significant financial toxicity around diabetes management experience more distress (47). A qualitative study of older adults with T1D using AID systems found that while participants experienced benefits to their diabetes management and quality of life from the technology, they also described weighing these benefits against the high costs and feeling ‘penalized’ financially due to the cost of devices (15). Thus, people who struggle to afford their devices may experience emotional distress around device use and navigating device issues, which could affect their supply.

Notably, frequently reported modifiable challenges to device use that people endorse are ways in which diabetes devices affect their self-perception, including disliking wearing devices, poor body image, and fear of stigma (16, 18, 19, 48, 49). These barriers highlight a fundamental challenge in one’s experience living with diabetes: accepting diabetes into one’s identity. ‘Identity’ refers to how one thinks of themselves, influenced by social networks, different environments, and personal experiences (50, 51). The process of integrating a chronic illness like diabetes into one’s identity is not always easy; people may struggle to accept diabetes and its treatment as part of their life and self, particularly when treatment for diabetes can be intrusive, affect perceptions of normalcy, and bring on judgment from others (52). Previous research suggests that more positive acceptance of diabetes and its treatment as part of one’s identity is associated with better glycemic and psychosocial outcomes (53, 54, 55).

There are many potential avenues to mitigate the emotional challenges to uptake and use of diabetes technologies. Structured education on devices, at initiation and as new challenges occur, can be valuable in reducing frustration and distress around device use (32, 56, 57, 58). Furthermore, it is imperative for clinicians to ensure that users have realistic expectations of what their devices can do (59, 60). There are also opportunities for patient-centered discussion and a teamwork approach to device uptake and use between patients and their providers. A qualitative study of racially/ethnically diverse young adults found that provider optimism about technology and tailored discussion of how technology would benefit the patient helped them feel more open and accepting of technology (61). Shared decision-making is also beneficial to device acceptance (61, 62), as this may help users to feel empowered in their informed decision to start technology. Additionally, given the known psychosocial impact of diabetes technologies (63), involvement of mental and behavioral health-care providers can be impactful in assisting people with adjusting and adapting to the emotional burdens of wearable technologies. Psychosocial care has been encouraged as a part of standard diabetes care (64) given the significant mental and emotional burden of diabetes management, suggesting that psychosocial care is applicable to technology use as well. There is also an opportunity to harness the concept of identity in encouraging device use. A recently developed measure of incorporation of diabetes into identity suggests three key processes in incorporation: stigma management, adjustment to perceived interference, and benefit-finding (55). Together, these concepts align with the commonly reported barriers to technology use. The process of incorporation is worthy of further study to investigate if and how addressing identity may be utilized to promote optimal, sustained diabetes device use.

## Perceptions and experiences of social consequences of device use

Wearing and using diabetes devices can bring up situations and challenges specific to social contexts with peers, at school, at work, and with family and other relationships. The large T1DX device barriers survey in adults and older adolescents found that around 10% of the respondents endorsed ‘Worries about what others will think of me’ and ‘I do not like diabetes devices because people notice them and ask questions about them’ (16). Further investigation via cluster analysis uncovered that those endorsing these specific social barriers tended to be younger adults with elevated diabetes distress (65). Similarly, in the T1DX study of adolescent-reported barriers to device use, 20% of adolescents endorsed worries about what others would think and 17% endorsed disliking devices because people would notice and ask questions about them (18). These findings are consistent with other research highlighting how adolescents and young adults feel that their T1D makes them unlike their peers, brings on negative judgment from others, or draws unwanted attention to themselves (52, 66, 67, 68, 69). It follows that having a device or devices worn on the body, that may be visible and audible to others, can contribute to concerns around stigmatization, which can make it more challenging to engage with devices in social situations (49).

Current technology also includes advanced features that may raise social concerns for the device user. For example, CGM systems allow for sharing data with one’s health-care team as well as with one’s social support system if they choose to use these features. Retrospective analysis of engagement data from over 26,000 CGM users with T1D has shown that a minority (38.7%) enable data-sharing features (70). This study did not explore factors contributing to sharing or not sharing data; however, other research has shed light on barriers that some CGM users experience when considering sharing data with a trusted person. If an individual with T1D is concerned that sharing their data with a spouse, partner, family member, or friend could add burden for that person, that concern may deter the individual from taking advantage of these features (20). Qualitative studies with youth and parents suggest that remote monitoring by parents is useful but could lead to increased frustration or conflict if data sharing results in excessive communication or involvement in diabetes care when the adolescent prefers to manage independently (25, 71, 72). Further, concerns about alarms being disruptive to the people around them, whether at home, at school, work, or elsewhere, may lead someone to silence or turn off alarms that could otherwise be helpful and contribute to improved diabetes management.

Efforts to promote sustained and optimal use of diabetes devices will need to attend to individual preferences for disclosing their diagnosis and/or engaging in social support in order to be effective. Discussion around disclosure of diabetes, particularly given the potential for devices to draw attention from others, may be valuable in helping people feel more control over others’ perceptions of devices and reduce perceived stigma. Tailored education may be beneficial in helping people understand how to customize alerts and help them feel empowered to proactively customize them to fit their life. In addition, enhancing problem-solving skills may help to work through specific social-related concerns, such as what to do if an alarm goes off at work or during an exam in school, or what to do if an alarm is disrupting someone else’s sleep overnight. Further, focusing on communication and self-advocacy skills may assist in thinking through whether to share data, with whom, and what the ‘ground rules’ and support preferences are for the person-sharing data. Evidence demonstrates that sharing data can be associated with a range of benefits such as collaborative diabetes management, prevention of severe hypoglycemia, and improved A1c (73). A pilot telehealth program involving older adults with T1D sharing data with a trusted care partner was found to be feasible and led to improved communication and peace of mind around diabetes (74). In addition, greater use of optional alert and notification functions (e.g. setting a high alert) has been linked to more optimal diabetes management and increased time-in-range (75, 76). In the large retrospective study of CGM use, while almost all (96–98%) CGM users with T1D enabled low, high, and urgent low alarms, fewer customized low (60%) and high (74%) thresholds (70). Therefore, to promote the optimal use of diabetes devices, tailored coaching and counseling may be effective for encouraging the use of some beneficial technology features within one’s own personal preferences for management within social settings.

Another important approach to addressing social-related concerns is through continuing advances in the design and functionality of diabetes devices. For example, diabetes technology that integrates with everyday smartphones and smartwatches is easier to engage with in social settings than a separate medical device. Further user-centered design approaches that engage device users in co-creating the design of devices and interfaces may assist with aligning with preferences to address social barriers (77). Increased visibility of diabetes devices, such as through representations in media, through more widespread use, and/or through connections with others with T1D who use devices may also help normalize the use of this technology which may then help individual users feel more comfortable adopting it with fewer concerns about standing out (78, 79).

## Practical challenges

Existing insulin pumps and CGM models all require attaching a device to the body, an experience that can be invasive both internally through the subcutaneous placement of a pump cannula or CGM sensor and externally through the physical and visible protrusion of a foreign object on the body. This reality comes with common associated barriers and hassles that many experience. A large T1DX device barriers survey found that issues related to wearing devices were most commonly endorsed after cost and insurance-related barriers (16). Specifically, 47% of survey respondents, who tended to be younger adults, said ‘hassle of wearing devices all the time’ got in the way of using devices, and 35% endorsed ‘do not like having diabetes devices on my body’ (16). The follow-up T1DX survey study with adolescents with T1D similarly found that wear-related issues were most common (59%) (18). While these data were collected prior to the arrival of the newest devices and systems, some constant features remain such as the need to use infusion sets for most insulin pumps and the need to have a CGM sensor adhered to the body. Some have pointed out that body image concerns may further impede the use of devices that must be worn on the body (30, 31). Qualitative studies have expanded upon the understanding of barriers and hassles associated with wearing devices on the body. For young children, challenges include painful insertion and having enough bodily real estate to place two devices on smaller bodies (24). Older adults and those with different physical abilities may experience different practical challenges in their use of diabetes devices; for example, arthritis or other conditions that limit or slow hand movements may make it difficult to use and hold small devices, while retinopathy and/or decreased visual abilities may affect how someone is able to view device interfaces (43).

In addition to the more consistent challenges of tolerating a device on the body, technology also imposes unexpected challenges on users. Diabetes devices are intended to be worn constantly, meaning they must go through the same activities and situations as the people who wear them. This may lead to issues with keeping the device on the body and working effectively (e.g. knocking a device off the body during sports). Technology is not perfect and can malfunction or require troubleshooting in real time, which can create stress for device users who rely on technology to help manage T1D (80). A recent survey of CGM users found that the majority experience problems with insertion (63.5%) or a device falling off (61%) (81). These experiences can lead to gaps in device use while waiting for replacement supplies from the device company, which may have negative impacts on diabetes management and overall health (81).

With devices worn on the body for long periods of time, dermatological reactions (including allergic contact dermatitis) remain significant concerns that are getting increased attention in the literature (82, 83, 84, 85, 86). Reactions have been noted with the use of adhesives in sensors and insulin infusion sets (IIS) as well as IIS catheters (87). Among many different substances and materials used in adhesives that have been linked with potential skin reactions, isobornyl acrylate has been identified as one such substance in CGM and insulin pump adhesives that can lead to allergic contact dermatitis (88). These types of skin reactions may dissuade someone from continuing to use a device due to discomfort, pain, and the frustration that comes with continued hassles of wear (89). One pediatric study found that despite the widespread experience of dermatological issues, skin-related quality of life impairments were infrequently endorsed (86). Still, someone who has experienced severe skin reactions in the past with one device may be less likely to be willing to try a different device in the future.

Device onboarding support, which is recommended by the American Diabetes Association’s Standards of Care (90), is important to provide new device users with the education and troubleshooting skills to incorporate diabetes technology into their daily lives (91, 91, 92, 98; https://www.dexcom.com/training-videos). In particular, support from a certified diabetes care and education specialist (CDCES) may be beneficial for working through some of the common practical and physical barriers that device users experience. Certified diabetes care and education specialists are recommended to have competency in optimal device use including placement, insertion techniques, and troubleshooting, as well as being able to support individualized decision-making for each person with T1D (94). Receiving ongoing support for device use can also help with addressing potential skin reactions. One approach described to mitigate dermatological reactions, and subsequent burden, is to use a hydrocolloid or silicone-based plate between the skin and the device to provide a protective barrier; however, it should be noted that the need to obtain these barriers requires additional cost of supplies (88). In the longer term, future advances in diabetes devices will likely aim to minimize the daily hassles and burdens associated with wearing and using devices (e.g. through smaller size, longer wear time, and fewer separate devices to carry). Ideally, devices may be designed with users of all ages and abilities in mind to ease the initial learning curve and support ongoing use (43, 95). At each stage in decision-making about device adoption and continued use, it can be helpful to frame the hassle/burden of wearing devices on the body within a larger context of the pros and cons of diabetes devices in one’s life. This framing provides each person the opportunity to make their own decision about what hassles and tradeoffs they are willing to experience to be able to receive the benefits (96).

## Summary

**Figure 1:**
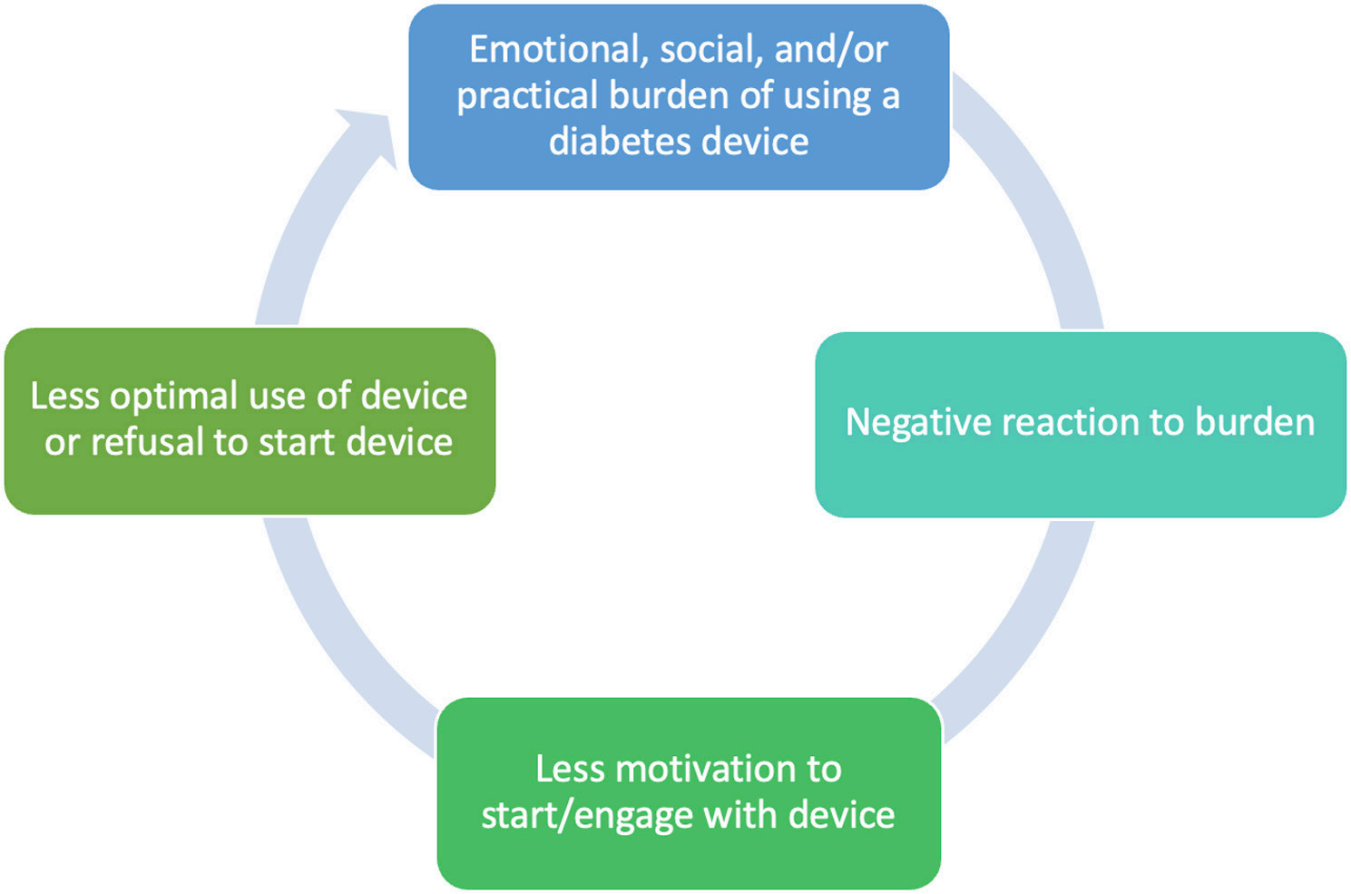
Negative cycle in which emotional reactions and experiences with devices may contribute to less optimal use and increased frustration and distress.

**Figure 2:**
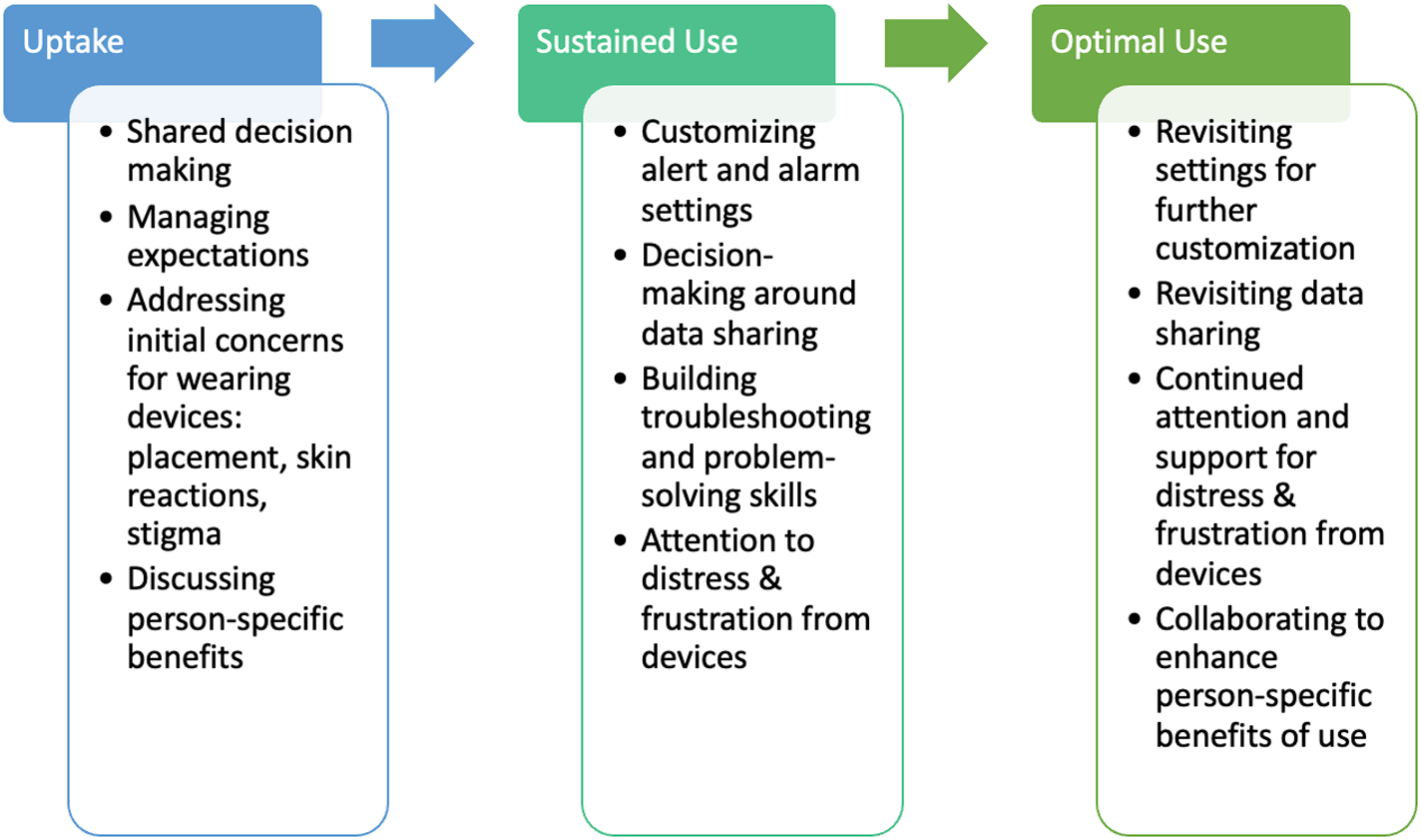
Example strategies to support each phase from diabetes device uptake to optimal use.

As diabetes technology continues to improve and advance, greater benefits can be experienced by people with T1D. However, it is important to contextualize these benefits within the device user’s lived experience with wearable devices (CGMs, insulin pumps, AID). Users may experience emotional, social and practical burdens, and hassles that may impede uptake, sustained use, and optimal use over time. We propose that emotional, social, and practical challenges associated with device use evoke strong emotional and/or cognitive reactions in users, which can affect their motivation, engagement, and decision to use diabetes technologies (Fig. 1). Without attention to these areas, device discontinuation is a risk (21). Attention to emotional distress, tailored education and skill-building for problem-solving and troubleshooting, and support for integrating device use into one’s life and social context should be critical components of programs to promote sustained and optimal use of diabetes devices over time (Fig. 2). Importantly, clinical support that is centered on the needs, preferences, and personal priorities of the person with T1D will ideally aid each person in choosing the right device(s), or not, to fit their life while maximizing benefits and minimizing burden. Given that diabetes devices must be worn on the body and interacted with daily, choosing whether and which one(s) to use is highly personal. Person-centered suggestions and decision-making will also support sustained use over time.

## Declaration of interest

MLT and PVC report no declarations.

## Funding

MLT is supported by National Institute of Diabetes and Digestive and Kidney Diseases of the National Institutes of Health (K23DK119470). The content is solely the responsibility of the authors and does not necessarily represent the official views of the National Institutes of Health.
